# Tumor Budding as a Useful Prognostic Marker in T1-Stage Squamous Cell Carcinoma of the Esophagus

**DOI:** 10.1002/jso.23341

**Published:** 2013-04-22

**Authors:** Hitoshi Teramoto, Masahiko Koike, Chie Tanaka, Suguru Yamada, Goro Nakayama, Tsutomu Fujii, Hiroyuki Sugimoto, Michitaka Fujiwara, Yasuhiko Suzuki, Yasuhiro Kodera

**Affiliations:** 1 Department of Surgery II, Nagoya University School of MedicineNagoya, Japan; 2 Department of Pathology, Tousei General HospitalAichi, Japan

**Keywords:** tumor budding, prognosis, esophageal cancer, squamous cell cancer, esophagectomy

## Abstract

**Background:** Establishing a new prognostic factor for early-stage cancer may seem difficult due to the small number of disease-specific deaths. Tumor budding has been recognized as a useful microscopic finding reflecting biological activity of the tumor.

**Methods:** Tumor budding stand for isolated single cancer cells and cell clusters scattered beyond the tumor margin at the invasive front. It was searched for in the resected esophagus with T1 squamous cell carcinoma (SCC), and the correlation between the tumor budding, patient survival, and various pathologic factors were analyzed to verify whether tumor budding is a prognostic factor in superficial esophageal cancer.

**Results:** Seventy-nine patients undergoing curative esophagectomy were assigned to frequent (n = 29) and rare (n = 50) groups according to the microscopically observed frequency of tumor budding in the tumor. Three-year survival rates after esophagectomy were 48.8% for the frequent group and 94.5% for the rare group. Multivariate analysis using the Cox proportional hazards model identified this morphological variable as a significant independent prognostic factor.

**Conclusions:** Tumor budding reflects the biological activity of the tumor and may be a useful prognostic indicator even in early-stage SCC of esophagus.

## INTRODUCTION

Squamous cell carcinoma (SCC) of the esophagus is more aggressive compared with other cancer of the gastrointestinal tract. In case of colorectal or even gastric cancer, T1-stage cancer is usually defined as an “early” cancer and is nowadays treated by limited surgery, resulting in both oncologically favorable outcome and preservation of quality of life. Some patients with T1-stage esophageal SCC, however, present with systemic disease at the time of diagnosis and are incurable even if treated by aggressive surgical approach. One could argue, therefore, that T1-stage SCC is not necessarily an “early” cancer and, after clinical staging and scrutiny of the resected specimens, the patients could actually be classified into a wide range of clinical stages, presumably depending on the aggressiveness of each cancer. One of important clinical questions regarding superficial esophageal cancer is how to select localized cancer which can be removed completely by endoscopic treatment 1,2. Sophistication in modern endoscopic technique enables complete removal of primary tumors invading as far as the upper-half of the submuscosa 4. Nonetheless, the endoscopic resection is currently recommended only for tumors confined to the mucosal lamina propria, a tumor stage known to have no risk of lymph node metastasis 1,2.

Microscopic finding of tumor budding, isolated single cancer cells and cell clusters scattered beyond the tumor margin at the invasive front, has originally been recognized as a sign of aggressive phenotype in colorectal adenocarcinoma 5,6. Authors have already reported that the finding of tumor budding also is a clinically useful prognostic factor for SCC of the esophagus 8. However, whether the presence or absence of budding could be applied to predict outcome of more superficial cancer remains to be proven. As a rule, pathologic findings that reflect aggressive biology are more likely to be observed in the specimens of advanced cancer. It follows that findings such as budding may be a rarity among T1-stage tumors in general. In the current study, the authors evaluated prognostic relevance of tumor budding exclusively with the specimens of T1-stage esophageal cancer, obtained by surgery or endoscopic mucosal resection (EMR). Given the diversity of outcome among patients with superficial SCC of the esophagus, search for a useful prognostic marker to identify patients who should be given multimodal therapy after surgery or those who need salvage treatment after EMR 1–9 is warranted even for this population. To be more specific, establishing a new method to predict lymph node involvement in patients with cancers invading only as far as the muscularis mucosa (m3) or superficial submucosal layer (sm1) is mandatory to expand the indication of EMR.

## METHODS

Four-hundred and three patients with SCC of the esophagus underwent esophagectomy between May 1988 and July 2005 at Department of Surgery II, Nagoya University Graduate School of Medicine. Of the 403 patients, 108 patients were confirmed pathologically to have T1-stage lesions. Of these, 22 patients who received preoperative treatment (chemotherapy and/or radiotherapy), 3 patients who underwent inadequate lymphadenectomy due to co-morbidities, and 4 patients with other coexisting cancer were excluded. The remaining 79 patients who underwent curative esophagectomy were analyzed. Table I shows all characteristics of 79 patients. All patients underwent follow up for 5 years (mean 85 months, range 78-166 months) or until death. In the meantime, the patients were followed monthly in the first year, every 2/3 months thereafter and were routinely administered computed tomography every 6 months. Thirty-two patients die in any cause, and of 18 patients were died by recurrence of esophageal cancer. Eleven patients received postoperative adjuvant therapy (chemotherapy and/or radiotherapy).

**TABLE I:** Clinical Characteristics of 79 Patients

Mean age (range)	62.2 (44–82)
Gender	
Male	67 (85%)
Female	12 (15%)
Location of the tumor	
Upper thoracic	6 (8%)
Middle thoracic	50 (63%)
Lower thoracic	20 (25%)
Abdominal	3 (4%)
Tumor depth^a^	
T1a	21 (27%)
T1b	58 (73%)
Lymph node status	
N0	23 (29%)
N1	56 (71%)
Stage of the lesion	
I	56 (71%)
II	17 (21%)
III	0 (0%)
IV	6 (8%)
LN dissection	
Two-field lymph dissection	60 (76%)
Three-field lymph dissection	19 (24%)
Adjuvant therapy	
None	64 (81%)
Chemotherapy	11 (14%)
Radiotherapy	8 (10%)
Both	4 (5%)

a T1a: Invasion into the lamina propria or muscularis mucosae but not beyond the muscularis mucosae, T1b: Invasion into the submucosapatients.

Six of these 79 patients had undergone EMR before radical surgery. These patients received additional esophagectomy because scrutiny of the endoscopically resected specimens revealed cancerous invasion to the muscularis mucosae or upper-third of the submucosal layer, which implicates risk for lymphatic spread and metastasis to the regional lymph nodes. Evaluation of tumor budding for these six patients was conducted with the EMR specimens, however, since no residual cancer was found in the esophageal mucosa of the surgically resected specimens. The other 73 patients underwent esophagectomy with 2-field or 3-field lymph node dissection as their first treatment.

**Fig. 1:**
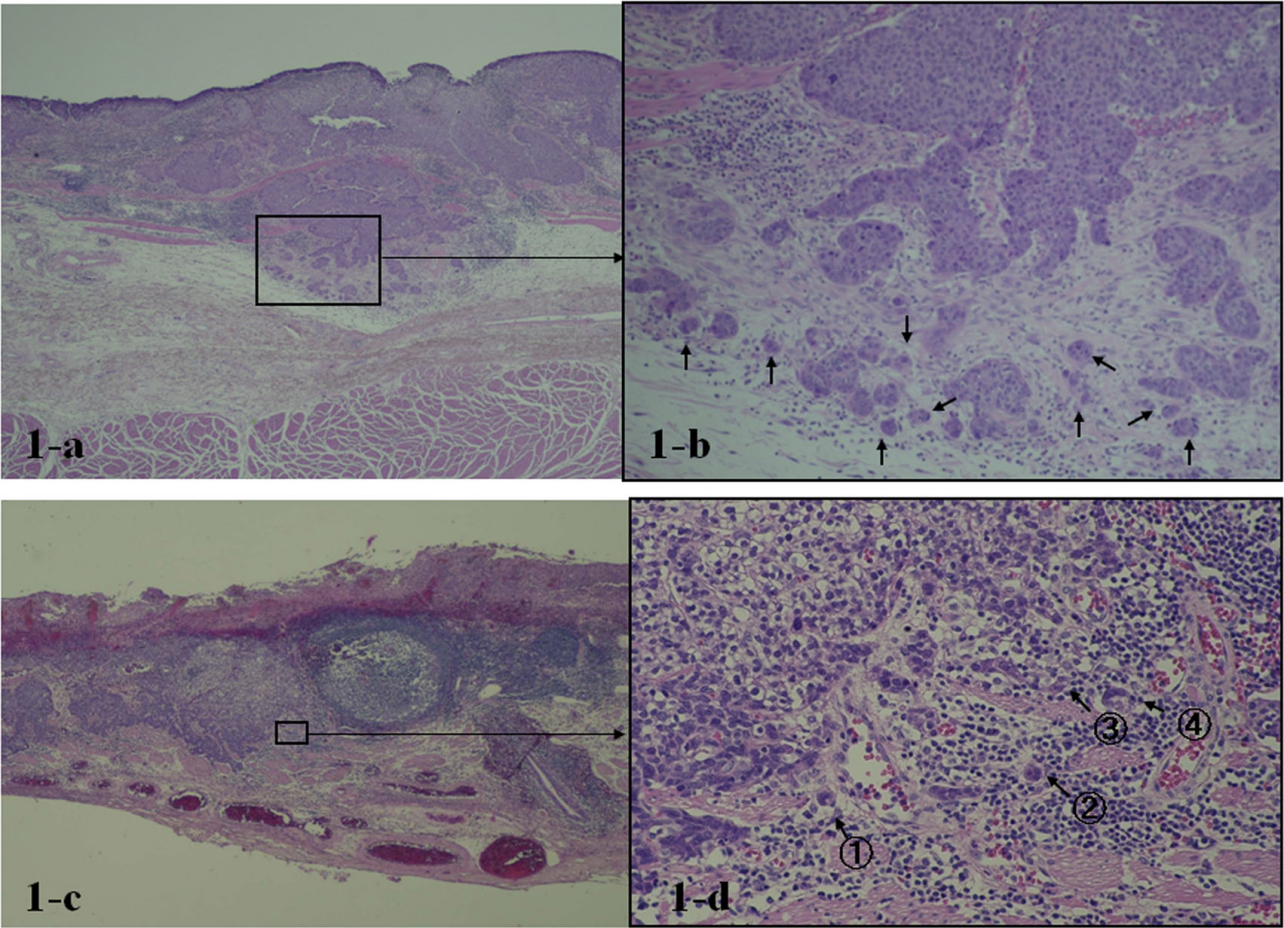
**a**,**b**: The configuration of a lesion with tumor budding in T1 esophageal cancer. (many tumor budding foci, showing by arrows; a, 100×: b, 400×). **c**,**d**: Tumor budding in an EMR specimen of T1 esophageal cancer (tumor budding foci, showing by arrows a, 5×; b, 400×).

Formalin-fixed and paraffin-embedded specimens were sectioned to a thickness of 3 µm and stained with the hematoxylin and eosin. Tumor budding at the invasive front was evaluated in addition to the routine pathologic findings defined by the Japan Esophageal Society guidelines 10. All specimens were observed independently by two observers (pathologist and authors). In case of a disagreement on the grading of pathologic findings, two observers reviewed slide together and reached a consensus diagnosis. Isolated single cancer cells and clusters composed of fewer than five cancer cells were defined as budding foci (Fig. 1a,b). These scattered foci were observed at the stroma in the active invasive front. To semi-quantify this finding, a field in which the budding intensity was considered as maximal was selected in the slide containing the deepest portion of tumor penetration, and the number of budding foci was counted in a field using 20× objective lens. Patients were classified into the following two groups based on the number of tumor budding; frequent group in which the budding intensity was ≥3, and rare group in which the budding intensity was <3.

Cumulative survival rates were calculated by the Kaplan–Meier method. The log-rank test was used to evaluate differences between survival curves. For comparing rare and frequent groups the chi-squared test and *t*-test were used. Several pathologic factors, including those associated with survival by univariate analysis, were subjected to multivariate analysis using Cox's proportional hazards model. Statistical calculations were performed with a personal computer using Statview J-4.5 software (SAS Institute, Cary, NC). *P* < .05 was considered as statistically significant.

## RESULTS

**Fig. 2:**
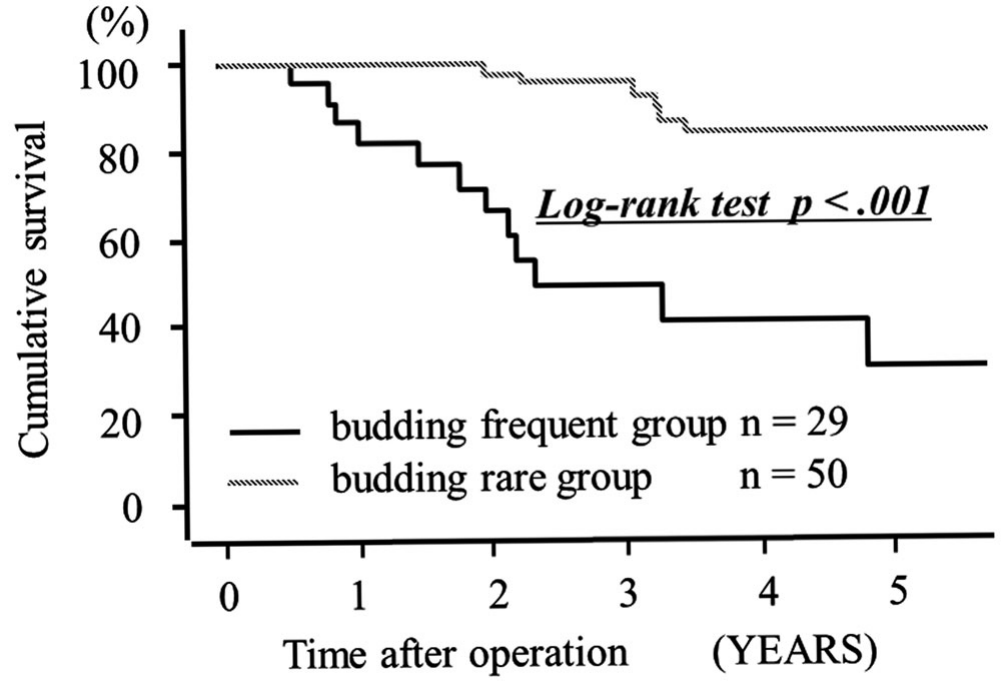
Cumulative curves for survival after esophagectomy in patients with frequent (n = 29) and rare (n = 50) budding. The difference in survival between groups was significant (*P* < 0.001).

There was no operative death. The mean number of budding foci in the specimens was 2.01 ± 2.61 (range: 0–10). Twenty-nine patients (36.7%) were classified into the frequent group and the remaining 50 patients fell into the rare group. Cumulative 3-year survival rate for the rare group (94.5%) was significantly higher than that for the frequent group (48.8%, *P* < 0.001; Fig. 2). However, no significant differences were observed between the two groups regarding gender distribution, age, and location of the tumors. Patients in the frequent group tended to have more advanced disease in terms of clinical stage than patients in the rare group. Consequently, three-field lymph node dissection and adjuvant chemotherapy and/or radiotherapy were more frequently delivered to patients in the frequent group.

Patients with lymph node metastasis, lymphatic vessel invasion, vascular invasion, infiltrative patterns of invasion to the surroundings (inf β and γ as defined by the Guidelines for Esophageal Carcinoma of the Japan Esophageal Society 10) and greater depth of invasion (T1b) were more commonly observed in the frequent group (*P* < 0.001, *P* < 0.001, *P* < 0.001, *P* < 0.001, and *P* = 0.003, respectively; Table II). Univariate analyses are shown in Table III. The univariate analysis showed that each of the following pathological factors had a significant influence on prognosis; tumor budding, lymph node metastasis, vessel invasion, depth of tumor, intramural metastasis, inf, lymph node dissection, adjuvant therapy. Multivariate analysis was performed using these pathologic factors as covariates, and tumor budding, lymph node metastasis, vascular invasion, and lymphatic vessel invasion were identified as significant independent prognostic factors (hazard ratio, 4.42, 4.55, 6.10, and 2.21, respectively; Table IV).

**TABLE II:** Pathologic Features of the Tumor

	Tumor “budding”		
		
	Frequent (n = 29)	Rare (n = 50)	*P*-value
Lymph node metastasis	17	6	< 0.001
Lymphatic invasion	24	13	<0.001
Vessel invasion	9	1	<0.001
Intramural metastasis^a^	2	0	0.131
inf(α/β and γ)^b^	5/24	34/16	<0.001
Tumor differentiation (well and moderate/poor)	22/7	46/4	0.0087
Depth of tumor invation (T1a/T1b)^c^	2/27	19/31	0.003
Tumor size (≤4.0 cm/<4.0 cm	21/8	42/8	0.253

a Metastasis, clearly separate from the primary tumor and located within the esophageal or gastric wall, should be recorded as intramural metastasis.

b Inf: the pattern of infiltration of a tumor, α: expansile growth of tumor nests forming a distinct border with surrounding tumor, β: intermediate pattern, between α and γ, γ: infiltrative growth of tumor nests forming an indistinct border with surrounding tissue.

c T1a: invasion into the lamina propria or muscularis mucosae but not beyond the muscularis mucosae, T1b: invasion into the submucosa.

**TABLE III:** Univariate Analysis of Pathologic Factors

		95% Confidence limits		
			
	Hazard ratio	Lower	Upper	*P*-value
Tumor “budding”				
Negative	1			
Positive	10.00	3.26	30.3	<0.001
Lymph node metastasis				
None	1			
Positive	7.40	2.77	20.00	<0.001
Vessel invasion				
No invasion	1			
Vein	6.49	2.26	18.87	<0.001
Lympatic vessel	5.29	1.74	16.13	0.003
Tumor differentiation				
Well/Mod	1			
Por	2.05	0.73	5.76	0.174
Depth of the tumor				
T1a	1			
T1b	8.93	1.19	66.67	0.033
Intramural metastasis				
Negative	1			
Positive	5.18	1.18	22.72	0.029
Inf				
α	1			
β/γ	2.99	1.06	8.40	0.038
Tumor size				
≤4.0 cm	1			
<4.0 cm	1.28	0.42	3.89	0.662
Lymph node dissection				
2F	1			
3F	3.77	1.49	6.67	0.005
Adjuvant therapy				
None	1			
Chemotherapy	5.110	1.90	13.73	0.001
Radiotherapy	3.93	1.39	11.06	0.009

**TABLE IV:** Multivariate Analysis of Pathologic Factors

		95% Confidence limits		
			
Variable	Hazard ratio	Lower	Upper	*P*-value
Tumor “budding”	4.42	1.08	18.18	0.039
Lymph node metastasis	4.55	1.07	19.23	0.04
Vessel invasion				
Vein	6.10	1.26	29.4	0.025
Lymphatic vessel	2.21	1.74	16.13	0.328
Tumor differentiation				
Well and Mod/Por	0.83	0.18	3.68	0.802
Depth of the tumor				
T1a/T1b	2.07	0.21	20.00	0.530
inf				
α/β and γ	1.63	0.41	6.41	0.487
Tumor size				
≤4.0 cm/<4.0 cm	1.40	0.35	5.65	0.633
Lymph node dissection				
2F/3F	1.44	0.40	5.18	0.576
Adjuvant therapy				
Chemotherapy	5.110	1.90	13.73	0.001
Radiotherapy	3.93	1.39	11.06	0.009

Twenty-six (36.7%) of 79 T1-stage esophageal cancer invaded the muscularis mucosae or upper-third of the submucosa (m3/sm1), and four (15.4%) of these patients had lymph node metastasis. Four of 26 patients with cancers invading the m3/sm1 layer belonged to the frequent group, of which two patients had lymph node metastasis. Six patients treated by EMR had been found to have lesions invading the m3/sm1 layer and underwent esophagectomy as a salvage treatment. One of these patients had four budding foci in the primary lesion and subsequently fell into the frequent group. In this patient, scrutiny of the specimen obtained by salvage surgery revealed lymph node metastasis (Fig. 1c,d).

## DISCUSSION

Tumor budding has been reported as a good prognostic indicator reflecting *malignant potential* in colorectal cancer 5,6. More recently, authors have reported tumor budding as an independent prognostic factor also for SCC of the esophagus, useful for decision making in clinical practice 8. SCC of the esophagus is one of highly aggressive cancers and even T1-stage cancer, which is localized at the primary site and is curable in case of other cancer types, often involves regional lymph nodes and is sometimes found to have developed into a systemic disease. Since treatment of T1-stage cancer could range from EMR to radical surgery, an accurate prognostic marker to define the degree of aggressiveness exclusively for this stage is essential for adequate decision making. On the other hand, it has been well accepted that malignant potential of cancer generally increases during disease progression, and pathologic findings reflecting aggressive phenotype is unlikely to be abundant in superficial cancer. Identification of relevant pathologic finding that predicts outcome was therefore estimated to be difficult when dealing exclusively with T1-stage cancer.

In reality, however, tumor budding was actually observed among primary tumors of T1-stage cancer, and were shown by multivariate analysis to be an independent prognostic factor for T1-stage SCC along with lymph node metastasis and vascular invasion. Tumor budding represents microscopic cluster of cancer cells scattered beyond the invasive margin. This finding indicates dissociation at the invasive front, which is considered the first step in metastasis of a solid tumor. It has been reported in case of colorectal cancer that these cells tend to be of undifferentiated phenotype with additional propensity to metastasize, but the degree of differentiation and its role in tumor budding or metastasis has not been explored in the current study 6–11. Nevertheless, since tendency to metastasize is one of crucial factors determining the aggressive nature of a tumor, it seems natural that the number of budding foci reflected outcome of patients with SCC. Because tumor budding can be evaluated easily using surgically resected or EMR specimens as a part of routine pathologic examination, clinicians may benefit promptly from the finding in the current study.

However, there actually is no standardized criterion to measure the degree of budding and classify patients accordingly, even for colorectal carcinoma. Methods to decide on the frequency of tumor budding had been ambiguous in the past, but a proposal by Ueno et al. to count the budding foci in a field where they are most frequently observed somewhat clarified the methodology 6. Even then, cutoff value of the number of budding foci remains elusive. Although the number of budding per a field of ≥5 was designated as frequent in the previous study, the cutoff value in the current study was brought down to 3. Since the mean number of budding foci observed in the current series was 2.01 ± 2.61 and was fewer than in the case of advanced cancer, the cutoff value had to be arbitrarily reduced to raise sensitivity. Further validation study with another set of samples is mandatory to establish a universally applicable cutoff value.

Most effective therapy for SCC of the esophagus remains complete surgical resection. Esophagectomy, however, is associated with significant morbidity and mortality 12 while exerting detrimental influence over the health related quality of life. EMR is ideal in that it is less invasive and is organ-preserving. However, only patients with superficial cancer without lymph node metastasis can expect complete cure by this treatment, and adequate patient selection remains an issue. In addition of to the EMR technique, recent development of endoscopic submucosal dissection (ESD) expanded the frontiers of endoscopic treatment 13. ESD enables resection of larger and more deeply infiltrated tumors with a sufficient surgical margin. As the technique for endoscopic resection becomes more sophisticated, adequate selection of patients who can be treated by endoscopic approach without salvage treatment becomes more complex and demanding. Cancer may involve the lymphatic when it invades as far as the muscularis mucosa, and nodal metastasis is reportedly observed in as many as 30% of the patients 2 when cancer invades the submucosa. On the basis of these findings, conventional indication of EMR is limited to carcinoma in situ and tumors invading only as far as the lamina propria 14. Although ESD can technically remove the primary tumors invading the upper-half of the submucosa 4, lymph node metastases occur in 10–15% of cases that invades the muscularis mucosae or upper-third of the submucosa (m3/sm1) 2–15, and these patients are indicated for radical surgery in order to raise the cure rate. In other words, more than 85% of patients with m3/sm1 lesion actually do not require additional treatment to dissect lymph node metastasis. Sensitivity and specificity of radiographic imaging studies for detection of nodal metastasis have not been found satisfactory 16. More accurate prediction of nodal metastasis through examination of the specimens resected by EMR or ESD is crucial if one is to avoid over surgery without sacrificing minority of patients with nodal metastasis.

To conclude, novel information that adds to the conventional clinical parameters such as depth of invasion or tumor diameter as a predictor of nodal metastasis is mandatory to expand the indication for less invasive endoscopic treatment for SCC of the esophagus. Tumor budding was shown to be an independent prognostic factor for T1-stage SCC along with lymphatic and venous invasion, and lymph node metastases were significantly more common among the budding frequent group. Evaluation of budding could be conducted as a part of routine histopathologic examination both with surgically resected and EMR specimens.
